# A Conjoint Exploratory Factor Analysis of Normative and Pathological Personality Traits in Older Adults

**DOI:** 10.1093/geroni/igaf122.3380

**Published:** 2025-12-31

**Authors:** Lisa Stone-Bury, Daniel Segal

**Affiliations:** Bucknell University, Lewisburg, Pennsylvania, United States; University of Colorado Colorado Springs, Colorado Springs, Colorado, United States

## Abstract

The Alternative Model of Personality Disorders (AMPD) is a dimensional model of personality disorders (PD) that measures PD symptoms according to a five-factor pathological personality trait model. This pathological trait model was designed to align with the Big Five model of normative personality traits: Negative Affect-Neuroticism, Detachment-Extraversion, Antagonism-Agreeableness, Disinhibition-Conscientiousness, and Psychoticism-Openness. Theoretically, lower-order facets of both models should overlap in these proposed directions to produce a five-factor model. This study examined this theoretical conjoint structure among community-dwelling older adults (n = 200) who were recruited online and completed the Personality Inventory for DSM-5 (PID-5; pathological personality traits) and the Big Five Inventory-2 (BFI-2; normative personality traits). Bivariate correlations indicated strong relationships in expected directions. An exploratory principal factor analysis with oblimin rotation was conducted between the PID-5 and BFI-2 facets. Model fit indices (KMO, Bartlett’s test, RMSEA) and commonalities of the scales indicated that the analysis could proceed. A six-factor model emerged: (1) Negative Affect-Neuroticism, (2) Detachment-Extraversion, (3) Antagonism-Agreeableness, (4) Disinhibition-Conscientiousness, (5) Psychoticism-obsessive, and (6) Openness. Essentially, four of the joint domains largely replicated as proposed in the AMPD (with some substantial differences between specific loadings). However, the proposed joint Psychoticism-Openness domain did not emerge, instead loading onto two separate factors (with substantial theoretical differences). Normative fluctuations in Big Five traits that occur across the lifespan may create difficulties when attempting to connect it to the AMPD. The present findings suggest that further revision to the Psychoticism domain is needed for the model to be appropriately applied to older adults.

